# The Existing Methods and Novel Approaches in Mycotoxins’ Detection

**DOI:** 10.3390/molecules26133981

**Published:** 2021-06-29

**Authors:** Edyta Janik, Marcin Niemcewicz, Marcin Podogrocki, Michal Ceremuga, Leslaw Gorniak, Maksymilian Stela, Michal Bijak

**Affiliations:** 1 Biohazard Prevention Centre, Faculty of Biology and Environmental Protection, University of Lodz, Pomorska 141/143, 90-236 Lodz, Poland; edyta.janik@unilodz.eu (E.J.); marcin.niemcewicz@biol.uni.lodz.pl (M.N.); marcin.podogrocki@biol.uni.lodz.pl (M.P.); leslaw.gorniak@biol.uni.lodz.pl (L.G.); 2 Military Institute of Armament Technology, Prymasa Stefana Wyszyńskiego 7, 05-220 Zielonka, Poland; ceremugam@witu.mil.pl; 3 CBRN Reconnaissance and Decontamination Department, Military Institute of Chemistry and Radiometry, Antoniego Chrusciela “Montera” 105, 00-910 Warsaw, Poland; m.stela@wichir.waw.pl

**Keywords:** mycotoxins, human health, chemical analysis, analitycal methods, food safety

## Abstract

Mycotoxins represent a wide range of secondary, naturally occurring and practically unavoidable fungal metabolites. They contaminate various agricultural commodities like cereals, maize, peanuts, fruits, and feed at any stage in pre- or post-harvest conditions. Consumption of mycotoxin-contaminated food and feed can cause acute or chronic toxicity in human and animals. The risk that is posed to public health have prompted the need to develop methods of analysis and detection of mycotoxins in food products. Mycotoxins wide range of structural diversity, high chemical stability, and low concentrations in tested samples require robust, effective, and comprehensible detection methods. This review summarizes current methods, such as chromatographic and immunochemical techniques, as well as novel, alternative approaches like biosensors, electronic noses, or molecularly imprinted polymers that have been successfully applied in detection and identification of various mycotoxins in food commodities. In order to highlight the significance of sampling and sample treatment in the analytical process, these steps have been comprehensively described.

## 1. Introduction

Mycotoxins are low molecular mass (MW ~700 Da) secondary metabolites of filamentous fungi which are harmful to human and animal health [1]. More than 400 different mycotoxins with various chemical structures and properties produced by a wide variety of fungal species, have been identified [2]. The main genera of mycotoxigenic fungi are: *Aspergillus*, *Fusarium*, *Penicillium*, *Alternaria*, *Claviceps,* and *Stachybotrys* [3]. Among the mycotoxins, aflatoxins (AFs), ochratoxin A (OTA), zearalenone (ZEA), patulin (PAT), fumonisins (FUMs), and trichothecenes (TCs) like deoxynivalenol (DON) and T-2 toxin (T-2) are the most concerning [4,5]. Many agricultural commodities such as wheat, barley, maize, oat, rice [2], vegetables, fruits [6] are contaminated with mycotoxins. Mycotoxins can also contaminate herbs [7,8], spices [9,10], and beverages like: wine, fruit juices, beer [11], and milk [12,13]. Different factors can influence processes of growth and production of mycotoxins in various fungi species. These include environment, temperature, humidity, water activity (aw), pH, nutrients, substrate nature, level of inoculation, physiological state, and microbial interactions [14]. Toxin formation can occur on the field, during processing, packaging, distribution, and storage of agricultural commodities or food processing [15]. The occurrence of mycotoxin contamination is more frequent in food and feed produced in developing countries due to their climate, poor production technologies, and crops storage conditions [16]. A large number of mycotoxins are chemically and thermally stable during food processing, including milling, boiling, baking, roasting, frying, and pasteurization [17]. Mycotoxins are characterized by a wide range of toxic properties. Depending on the dose or exposure duration, severe immediate reactions or long-term effects may occur [3]. Mycotoxins are associated with toxicities, such as hepatotoxicity [18], nephrotoxicity [19], genotoxicity [20], neurotoxicity, and immunosuppression [21]. Moreover, the International Agency for Research on Cancer (IARC) classified aflatoxin B1 (AFB_1_) in Group 1 (carcinogenic to humans), and OTA in Group 2B (possibly carcinogenic to humans) [22]. Due to these facts, their presence in food can pose a risk to human health and life. However mycotoxins are natural contaminants and their presence in food are unavoidable [23].

National and international institutions and organizations, like the US Food and Drug Administration (FDA), the European Commission (EC), the Food and Agriculture Organization of the United Nations (FAO), and the World Health Organization (WHO), have identified potential health hazards to humans and animals associated with food- or feed-borne mycotoxin intoxication and tackled this problem by developing regulatory limits for main mycotoxin classes and selected individual mycotoxins [24]. The FDA has prepared guidance documents [25] and booklet lists [26] for mycotoxins such as DON and AFs in food and feed. The EC has compiled comprehensive regulations regarding the maximum level for mycotoxins in different foodstuff [27]. The FAO has developed extensive worldwide regulations for mycotoxins in food and feed [28]. Additionally, in the eighty-third report of the Joint FAO/WHO Expert Committee on Food Additives fumonisins and AFs as food contaminants have been widely evaluated in terms of their toxicology, exposure, and daily limits [29].

All of these efforts to establish mycotoxins limits and standards have induced the development of various analysis methods for the identification and quantification of mycotoxins in food samples [24]. Methods that have been validated and applied in the analysis of mycotoxins in agricultural commodities include: chromatographic techniques, immunoassay-based methods, or rapid strip screening tests [30]. Although great progress has been made in this area, there are still significant challenges and disadvantages to these analytical methods that should be addressed. The chemical diversity and co-occurrence of mycotoxins, their different concentrations in agricultural products and complex food matrices with mycotoxin contamination require special extraction, clean-up, and detection methods [31]. Continuous improvements in mycotoxin analytical methodology are needed to comply with mycotoxin legislation, restrictions, and to protect consumer health and support the agriculture [32].

This review summarizes the used methods and novel innovative techniques applied for mycotoxins detection and analysis in a variety of foods. In addition, a brief presentation of extraction methodologies and clean-up procedures are included.

## 2. Food Sampling and Sample Preparation

The determination of mycotoxins in food samples is preceded by several different steps such as sampling and sample preparation. Sample preparation includes extraction and clean-up. Both are crucial and cannot be separated from each other. Appropriate performance of all these steps enables a proper mycotoxins ascertainment [33].

### 2.1. Sampling

Sampling is significant in determination of mycotoxin levels because mycotoxigenic fungi do not grow even on the substrate and it is difficult to obtain a representative bulk sample. Moreover, the existing mycotoxin contaminations in natural samples are not homogeneous [30]. Therefore, apart from liquid food samples such as milk or certain highly processed foods like peanut butter, traditional food sampling methods are usually not suitable for mycotoxin analysis [34]. In order to standardize the sampling procedures for mycotoxin analysis, Commission Regulation (EC) No. 401/2006 [35] specified the sampling and analysis methods for the official control of the mycotoxins levels in foodstuffs [36]. The example is the cereals and cereal products sampling method for lots <50 tons, where sampling plan shall be used with 10 to 100 incremental samples, depending on the weight, resulting in an aggregate sample of 1 to 10 kg [35]. Inadequate sampling is associated with errors in the evaluation of the mycotoxin level of the lot could easily occur, usually leading to an underestimation. If sampling is performed for monitoring/surveillance purposes, poor sampling could give false information for risk assessors/managers. For inspection purposes, incorrect sampling can cause problems in litigations [37].

### 2.2. Grinding and Mixing

In order to accelerate the chemical reaction process of extraction and to increase the chances to detect the mycotoxins, the sample should be ground to the final particle size of approximately 500 µm opening size and homogenized to whole wheat flour or powder-like consistency [35,38]. Once homogeneity is obtained, the sample should be mixed. According to the conducted research and techniques comparison, the slurry mixing process appears to be a good option. This process resulted in very small particles size and consequently homogeneous samples with the lowest variation ratio [39].

### 2.3. Extraction and Purification

Extraction from contaminated food and feed samples aims to remove mycotoxins from the sample using appropriate solvents. It is the first step of sample preparation. QuEChERS (Quick, Easy, Cheap, Effective, Rugged, and Safe) method was initially developed for pesticide analysis, but it facilitates a simultaneous detection of different groups of mycotoxins in various matrices [40]. This method firstly requires an extraction with acetonitrile water, followed by liquid–liquid partitioning induced by the addition of inorganic salts. As a consequence, some polar components of the matrix remain in the aqueous layer, while mycotoxins are moved into the organic phase. Next, a dispersive solid phase extraction is applied to reduce other matrix compounds from the organic phase [41]. QuEChERS has been used for the analysis of different mycotoxins in numerous food matrices such as OTA, AFs, and citrinin in eggs [42], OTA and AFs in cereals [43], and in berries-derived jam and juice [44]. Next extraction method is liquid–liquid extraction (LLE) that is based on the different toxin solubility in aqueous phase and in immiscible organic phase. The compound extraction is placed into one solvent leaving the remainder of the matrix in the other [45]. LLE has been applied for the simultaneous analysis of AFs and OTA in breast milk [46]. Liquid–solid extraction (SLE) is a simple method for the mycotoxin’s extraction from solid matrices of various consistency. The extraction is based on the weighing of homogenized sample, and adding the extraction solvent, followed by agitating it in a shaker [47]. It has been confirmed that this method can be used to extract various mycotoxins from cereals [48]. Pressurized Liquid Extraction (PLE), also known as accelerated solvent extraction (ASE), is the same method as SLE but performed under increased pressure and temperature in a pressure-resistant vessel [49,50]. In these methods, conventional solvents at high temperatures (100–180 °C) and pressures (1500–2000 psi) are used to improve the extraction of analytes from the matrix [51]. PLE has been used to detect mycotoxins produced by *Alternaria alternata* in a tomato sample [50]. The next method is the Supercritical Fluid Extraction (SFE). SFE can minimize and eliminate the use of organic solvents by application of supercritical CO_2_. The SFE procedure is mostly used for the extraction of non-polar organic molecules [47] and has been carried in ZEA detection in maize flour [52]. All extraction methods, solvents, advantages, and disadvantages are summarized in Table 1.

**Table 1:** Extraction methods, solvent, advantages, and disadvantages.

Method	Solvent	Advantages	Disadvantages	References
QuEChERS	Acetonitrile, acetonitrile/acetic acid, acetonitrile/citric acid, acetonitrile/formic acid	Fast, simple, economical, reproducibility and applicability	Low enrichment factor in extracts of lipophilic compounds and the need for original modifications of the procedure	[53,54,55]
LLE	Hexane, cyclohexane	Effective for small-scale preparations	Does not provide a sufficiently clean analyte in all cases, time-consuming, possible loss of sample by adsorption onto the glassware	[45,49,56]
SLE	Acetonitrile/water, methanol/water	Smaller volumes of solvent	SLE alone can be not satisfactory to extract some mycotoxins without interference and additional purification steps are usually needed	[47,49]
PLE	Acetonitrile/water, acetonitrile/methanol	Extraction process can be automated, higher extraction efficiency in shorter time, lower amount of extraction solvent	High instrument price	[47,49,57]
SFE	supercritical CO_2_ fluid, acetonitrile	Fast, small solvent volumes, extraction of temperature sensible analytes	Low recoveries, high concentrations of co-extracts, high costs	[45,49]

Extraction is required to release the mycotoxins from the matrix. Clean-up of the extract is crucial to reduce matrix effects and eliminate substances, which can interfere with the next mycotoxin detection. Purification of the extract increases specificity and sensitivity, resulting in improvement of quantification accuracy and precision. The most commonly used methods for mycotoxins clean-up are solid phase extraction (SPE) and immunoaffinity columns (IAC), because they are rapid, efficient, and reproducible with a wide range of selectivity [34,51]. The SPE method involves the solid absorbents (where the mycotoxins are absorbed), which are usually packed in cartridges and rinsed in order to remove contaminants and capture the mycotoxins [58]. SPE is a rapid, efficient, and reproducible technique, but it presents some limitations, like the inability to use a single cartridge for all mycotoxins detection. Moreover, efficiency can be affected by several conditions, such as: the type of solvent, or the pH and ionic strength of the sample [59]. For commercial SPE, octadecylsilyl (C18), hydrophilic–lipophilic balance (HLB), amino-propyl (NH_2_) and silica gel can be used as adsorbent, but the majority of the commercial cartridges are not appropriate for high-throughput screening for multiclass mycotoxins [60,61]. Recently, carbon nanomaterial and magnetic carbon nanomaterial have been applied as alternative sorbent due to their strong absorption capacities. Among them, multi-walled carbon nanotubes (MWCNTs) were used for simultaneous determination of type A trichothecenes in rice, maize, and wheat [62]. What is more, multi-walled carbon nanotube-magnetic nanoparticles (MWCNT-MNPs) were introduced as sorbents for purification of ZEA in maize [63] and type A trichothecenes in coix [64].

In the case of IAC, monoclonal antibodies are used for certain mycotoxins detection. The target mycotoxin in the extract is bound by specific antibodies on the column during the sample flow through the column. At the same time, water-soluble impurities are removed during column washing and the mycotoxins are eluted from the IAC with pure methanol or acetonitrile for the following detection. IACs are very sensitive, selective, and can serve as universal and valid purification tool for tracing the mycotoxins. Furthermore, it is a user-friendly and solvent-saving method because of the antibodies’ specificity [65]. However, some limitations are linked with this technique. Columns have a limited ability to absorb mycotoxins and if the contents of mycotoxins in the sample exceed the column binding capacity, the mycotoxin is not effectively capture and bound, resulting in unreliable results. What is more, the numerous components in the matrix can interfere with the antibodies [66]. Other disadvantages include organic solvents, which can denature or devitalize the antibodies leading to difficulties in the reuse of IACs. Moreover, the operating costs of this method are substantially high [65]. IAC has been successfully applied in simultaneous analysis of OTA, ZEA, and AFs in wheat bran [67], OTA, AFs, and *Fusarium* toxins in maize [68] and cereals [69].

## 3. Techniques Used in Detection and Analysis of Mycotoxins

Since the discovery of the first mycotoxins, many different methods have been tested and used to analyze mycotoxins presence in food and feed [70]. The dominance of chromatographic techniques is observed, mainly due to the use of many different types of chromatography: thin layer chromatography (TLC) and high performance liquid chromatography (HPLC) in combination with various detectors such as diode array, fluorescence, and UV. Liquid chromatography-tandem mass spectrometry (LC-MS/MS) and gas chromatography-tandem mass spectrometry (GC-MS/MS) have been also widely applied in mycotoxin detection [30,71]. When rapid mycotoxin analysis is required, immunoassay methods such as enzyme-linked immunosorbent assay (ELISA) [72,73] and lateral flow immunoassay (LFIA) [74,75] are also important. Biosensors also appear to be a useful tool for identifying mycotoxins in food [76,77].

### 3.1. Chromatography Techniques

#### 3.1.1. TLC

A popular method of mycotoxin detection is TLC, which offers the possibility of economical screening of a large numbers of samples [78]. TLC is comprised of a stationary phase made of either alumina, silica, or cellulose, immobilized on an inert material like plastic or glass, which serves as a matrix. The mobile phase consists of methanol, acetonitrile, and water mixtures, that carry the sample in the solid stationary phase [79]. This method is effective for mycotoxins detection and some examples are listed in Table 2. Due to low costs, simplicity and fluorescent spots under UV light, it plays an important role in the analysis of many mycotoxins. This technique was developed for mycotoxin qualitative [80,81] and quantitative analysis [82,83,84]. However, TLC has low sensitivity and poor accuracy, which makes quantification very demanding [85]. Moreover, one of the main requirements is the sample preparation and the type of clean-up procedure, which strongly depends on the properties and the type of a mycotoxin [78].

#### 3.1.2. Liquid Chromatography (LC)

In order to overcome the limitations of the TLC technique, like limited plate height or humidity and temperature effects, the LC methods have been developed [85]. LC enables the simultaneous determination of several mycotoxins, regardless of their chemical structure and biological activity. An analytical column and a mobile phase are used for separation between analytes and the matrix components. What is more, it is applied as a separation and determination technique for high polarity, non-volatile, and thermally labile mycotoxins [53].

Mycotoxin analysis heavily relies on HPLC with different adsorbents, depending on the mycotoxin physical and chemical structure. Majority of the protocols used in mycotoxins detection are very similar. The most common detectors used in HPLC are the UV-visible (UV) or fluorescent (FLD) ones, which rely on the presence of a chromophore in the molecules but also on MS (single mass spectrometry, and tandem MS (MS/MS)) [45]. Some toxins already have a natural fluorescence (e.g., AFs, OTA) and can be detected directly in HPLC-FLD. HPLC-FLD is most commonly used for the detection of OTA in various matrices e.g., rice [86]. For other types of mycotoxins, such as fumonisin B_1_ (FB_1_), which do not possess chromophores in their structure, derivatization is necessary [30]. Derivatization is used to add chromophores or fluorescent moieties to the analyte. The process can be performed either before the chromatographic analysis (precolumn derivatization) or after the column separation and before detection (post column derivatization) [87]. The main limitations of HPLC technique are portability and practical issues based on the matrix effect, sample type and preparation, and also calibration [85]. The use of HPLC in mycotoxin analysis has been described in many publications, which is summarized in Table 2.

The use of LC-MS/MS for the determination of low molecular weight contaminants and residues at trace levels has increased significantly over the past two decades. MS/MS in combination with LC provides better sensitivity and reliability. Therefore, LC-MS/MS is a good standard tool to deal with the analytical challenges, which exist in food and feed safety chemical analysis, both in research and in commercial investigation [88]. LC-MS/MS provides high selectivity and sensitivity, greater certainty of analytes identification and a wider range of matrices compared to traditional methods using conventional detectors [89]. Majority of mycotoxigenic fungi can produce several distinct mycotoxins simultaneously. Therefore, agricultural commodities can be simultaneously contaminated by different mycotoxins [90,91]. Studies have confirmed that, the LC-MS/MS provides one of the most reliable and sensitive results for simultaneous determination of multi-mycotoxins analysis [92,93,94,95]. The examples are summarized in Table 2.

#### 3.1.3. Gas Chromatography (GC)

GC depends on differential partitioning of analytes between the two phases of GC column. The various chemical components in the sample distribute themselves between the stationary and mobile phases. After the separation process, volatile products are detected using a mass spectrometer, an electron capture detector (ECD) or flame ionization detector (FID) [85]. GC is rarely used in the mycotoxins’ analysis due to the low volatility and high polarity of analytes. Furthermore, the derivatization step is required for their conversion in volatile derivatives [34]. However, the GC-MS/MS method has been used for mycotoxins detection in milled grain-based products [96] and wheat semolina [97]. The examples are also listed in Table 2. The technique is highly sensitive and specific to mycotoxins and can be derivatized to a compound, which is sufficiently volatile for use in gas chromatography. The major problems in mycotoxin GC analysis are: column blockage, drifting responses, cross contamination from earlier samples and nonlinearity of calibration curves in some types of detectors [85].

**Table 2:** Chromatography techniques used in mycotoxin detection.

Technique	Mycotoxin	Food Commodity	LOD	LOQ	References
TLC	PAT	Apple juice	14 µg/L	-	[98]
TLC	AFB_1_	Herbs	0.01 µg /mL	-	[99]
TLC	AFs	Brazil nuts	-	2000 µg/kg	[83]
HPLC	DON	Wheat bran	12.58 µg/kg	-	[67]
HPLC	OTA	Wheat bran	0.40 µg/kg	-	[67]
HPLC	OTA	Wine	0.09 μg/L	-	[100]
HPLC	ZEA	Wheat bran	6.74 µg/kg	-	[67]
HPLC	AFB_1_	Peanut	0.10 µg/kg	-	[101]
HPLC	ZEA	Wheat flour	0.10 µg/kg	-	[101]
LC-MS/MS	AFs	Walnut kernel	0.004–0.013 µg/kg	-	[102]
LC-MS/MS	AFB_1_	Animal feed	0.72 µg/kg	-	[94]
LC-MS/MS	FB_1_	Maize	1 µg/kg	-	[94]
LC-MS/MS	T-2	Beer	0.001 µg/mL	-	[95]
LC-MS/MS	DON	Red wine	0.001 µg/mL	-	[95]
LC-MS/MS	AFB_1_	Cow milk	0.00002 µg/mL	-	[93]
LC-MS/MS	ZEA	Cow milk	0.00051 µg/mL	-	[93]
GC-MS/MS	T-2	Wheat-based cereals	-	5 µg/kg	[96]
GC-MS/MS	PAT	Rice-based cereals	-	10 µg/kg	[96]
GC-MS/MS	ZEA	Maize-based cereals	-	10 µg/kg	[96]
GC-MS/MS	DON	Wheat semolina	-	1.25 µg/kg	[97]
GC-MS/MS	DAS	Wheat semolina	-	5 µg/kg	[97]

### 3.2. Rapid Technologies

#### 3.2.1. ELISA

In addition to the sensitive but complex and costly chromatographic techniques, immunochemical methods such as ELISA are fast and simple screening techniques for on-site mycotoxin analysis [16,103]. ELISA is simple in design, enables simultaneous testing of multiple samples and its detection is precise [72,104]. It is a high-throughput assay with low sample volume requirements and less clean-up procedures compared to chromatographic methods such as HPLC or TLC [85]. The test is based on the interaction of the antigen-antibody complex with the presence of chromogenic substrates. The measurable result is obtained by spectrophotometric assessment [105]. ELISA technique has been widely used in mycotoxins detection in various types of food, as summarized in Table 3. Solcan et al., have also used ELISA to determine residues of AFB_1_ from chicken liver samples [106]. However, this technique has some disadvantages. Compounds with similar chemical groups can interact with the antibodies. The result of matrix effect or matrix interference that occurs in ELISA method may lead to under- or overestimation of mycotoxin concentrations in tested samples [107]. Moreover, inadequate ELISA validation, limits the technique to the matrices for which they have been validated [108]. Therefore, a comprehensive study of ELISA accuracy is needed for a wide range of food commodities [109].

**Table 3:** ELISA method used in the detection and of mycotoxins in various types of food.

Type of ELISA	Mycotoxin	Food Commodities	LOD	References
Direct ELISA	AFB1	Wheat	0.05 µg/kg	[108]
	AFB2		0.04 µg/kg	
	AFG1		0.06 µg/kg	
	AFG2		0.07 µg/kg	
Competitive ELISA	OTA	White tea	3.7 µg/kg	[110]
		Red tea	3.7 µg/kg	
		Spearmint	1.1 µg/kg	
	ZEA	White tea	8.3 µg/kg	
		Red tea	4.5 µg/kg	
		Spearmint	2.1 µg/kg	
Competitive ELISA	FUMs	Maize	30 µg/kg	[111]
	DON		70 µg/kg	
Green ELISA based on the SSB-assisted aptamer	AFB1	Corn	0.112 µg/L	[112]
	OTA		0.319 µg/L	
	ZEA		0.377 µg/L	
Competitive ELISA	OTA	Corn	1.9 ppb	[113]
		Barley	2.8 ppb	
		Wheat	3.5 ppb	
		Green coffee	3.3 ppb	
		Soybeans	2.5 ppb	

#### 3.2.2. Lateral Flow Immunoassay (LFIA)

LFIA, also called immunochromatographic strip test is a membrane-based immunoassay and works as a competitive method, using a labeled antibody as a signal reagent [114]. In the test, capillary beds, like pieces of porous paper drive the analyte and specific recognition elements bind moieties immobilized on the membrane surface [115]. The accuracy of LFIA mostly depends on signal labels. Traditionally, gold nanoparticles (GNPs) are the most widely used label to generate visual signals [116]. Besides nanoparticles, other materials such as magnetic nanoparticles (MNPs) [117], carbon nanoparticles (CNPs) [118], gold nanoparticles (AuNPs) [119], or quantum dots (QDs) [120] have been used as labels. Examples of different labels used for mycotoxins detection are presented in Table 4.

**Table 4:** Different labels used in mycotoxins detection and their sensitivity.

Label	Mycotoxin	Food Commodity	Sensitivity	References
GNPs	CPA	Rice Maize	1 μg/kg 2.5 μg/kg	[119]
GNPs	DAS	Rice	50 µg/kg	[121]
GNPs	FB_1_	Cereals	5 µg/L	[122]
ACNPs	ZEA T-2 DON	Maize	1 µg/kg 13 µg/kg 20 µg/kg	[116]
CdSe/ZnS QDs + GNPs	FUMs	Maize	62.5 µg/kg	[120]
CdSe/CdS/ZnS QDs	FB_1_+ FB_2_	Maize	2.8 µg/L	[123]

LFIA has many advantages, such as simplicity, fast results, and low cost, and is suitable for large-scale on-site screening. Moreover, sample clean-up can be omitted [124]. The main limitations of LFD are the interferences that may occur. What is more, it is a complicated matrix for the determination of trace analytes [125].

#### 3.2.3. Biosensors

In general, biosensors contain biological or biologically derived sensing element to detect specific bio-analytes integrated with a transducer in order to convert biological signal into an electrical signal [126]. Different types of transducers can be used for mycotoxin detection, including electrochemical (potentiometric, amperometric, and impedimetric), optical (surface plasmon resonance-SPR and fluorescence) and piezoelectric (quartz crystal microbalance-QCM) [127]. Commonly recognized materials are nucleic acids, peptides, enzymes, antibodies and cells, but other bioinspired elements like recombinant antibodies, aptamers, and molecularly imprinted polymers (MIPs) can also be used [128,129]. Furthermore, to improve the biosensors sensitivity, a wide variety of metal nanoparticles, carbon nanotubes (CNTs), nanofibers, and QDs are used due to their biocompatibility, physicochemical properties, and high surface volume ratio [130,131]. Various biosensors have been developed for different mycotoxins’ detection and are listed in Table 5.

The electrochemical biosensors are based on potentiometric, amperometric, and impedimetric detection methodologies [132]. The potentiometric sensor requires two (working and reference) or three (working, reference, and counter) electrode systems, and recognition event is provided by the changes in the circuit potential between working and reference electrodes [126,133]. The amperometric sensor, similarly to potentiometric requires two- or three-electrode system. The identification of an analyte by amperometric transducer is provided by the calculation of current data generated after the reduction and oxidation of electroactive species immobilized on the working surface after setting an appropriate potential [127]. Electrochemical impedance spectroscopy (EIS) method monitors the alterations that occur in the interface between electrode surface, modified by a nanostructured platform in contact with redox probe [134].

High sensitivity and real-time analysis are the main advantages of optical biosensors [135]. SPR and fluorescence approaches like fluorescence resonance energy transfer (FRET), are the main methods used [127]. The SPR system utilizes a thin metal (silver or gold) film between two transparent media with different refractive indices, like glass prism and sample solution. The SPR method detects alterations in the surface layer refractive index in contact with the sensor chip [136]. In the FRET system, the energy is transferred from excited donor fluorophore to nearby acceptor species [137]. The acceptor and donor in the FRET can be designed in biunique or one-to-multiple manners, ensuring the simultaneous application of multiple mycotoxin detection [127].

The QCM transducer consists of thin gold-plated crystal quartz, where electrodes are placed. A molecular recognition and binding event in the electrode surface lead to mass alteration and specific vibrations, when electric signal is sent by the quartz, which results in inducing alterations in the resonant frequency [127,138].

**Table 5:** Examples of biosensors used in different mycotoxin detection.

Recognition Element	Transducer/Technique	Mycotoxin	Food Commodity	Detection Limit	References
Antibody	Piezoelectric/QCM	AFB_1_	Peanut	0.83 ng/kg	[139]
Antibody	Piezoelectric/QCM	OTA	Red wine	0.16 ng/mL	[140]
Antibody	Impedimetric/EIS	AFB_1_	Corn	0.05 ng/mL	[141]
Antibody	Optical/SPR	OTA	Coffee	0.05 ng/mL	[142]
Aptamer	Impedimetric/EIS	PAT	Apple juice	2.8 ng/L	[143]
Aptamer	Optical/FRET	T-2	Wheat, maize	0.00093 ng/mL	[144]
Antibody	Amperometric/CV/DPV	ZEA	Maize	0.00017 ng/mL	[145]
Aptamer	Impedimetric/EIS	FB_1_	Maize	2 pM	[146]
Black phosphorene	Potentiometric/DPV	OTA	Grape juice, red wine	180 ng/mL	[147]

Rapid mycotoxin analysis share many common advantages, including speed, low costs, simplicity, and easy to use [109]. Important aspects include portability and multi-toxin detection. Mobility is also significant in face of the growing demand for on-site testing, which can take place, for example, on site of the food production process. The results are obtained relatively quickly, as the samples do not need to be shipped and analyzed at laboratories. It also prevents slowing down the food production process. Multi-toxin detection eliminates the need to perform multiple single-toxin tests for one sample batch [148]. The main limitations of these methods are matrix interference, antibody cross-reactivity, and the necessity of matrices’ validation [85].

### 3.3. Novel Technologies of Mycotoxins Analysis and Detection

In addition to the standard methods described above, there are several other methods that have been developed and may be useful in mycotoxin detection. Nevertheless, these methods have limited applicability and have not been widely used outside the research areas. Moreover, they require further verification and validation by recognized organizations such as the Association of Official Analytical Chemists (AOAC), International Organization for Standardization (ISO), or the European Standardization Committee (CEN) [34].

#### 3.3.1. Electronic Nose

An electronic nose (e-nose) consists of a range of nonspecific chemical detectors, which capture different volatile organic compounds (VOCs) and detects qualitative volatile fingerprints of toxigenic fungi. After achieving a fingerprint, detection of the odor gives preliminary information about the category of the produced metabolites by a pattern recognition system [149]. E-nose technology for the fungal infection detection is based on identifying specific VOCs related to the growth of fungi on cereal grains. The growth and biochemical pattern of mycotoxigenic fungi species cause chemical changes in the VOCs’ composition and a correlation between VOCs and mycotoxin concentration in food can be observed [150]. The e-nose has been successfully used for detection of OTA in dry-cured meat [151], AFs and fumonisins in maize [150] and DON in wheat bran [152] and in durum wheat [153]. In order to achieve wide usage of e-nose in the detection of mycotoxins, optimization for the quantification of low levels of mycotoxins in food samples is necessary. Moreover, the majority of mycotoxins are non-volatile organic compounds that pose a problem for e-nose detection [34].

#### 3.3.2. Fluorescent Polarization

**Figure 1:**
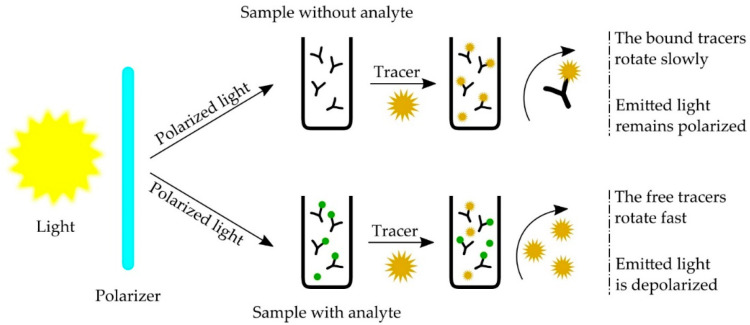
Schematic diagram of fluorescence polarization immunoassay.

Fluorescent polarization (FP) immunoassay is a method based on the competition between the analyte and the tracer (fluorophore labeled analyte) for specific antibody-binding sites. The binding of the tracer to the antibody has an impact on the rotation of the tracer molecule, increasing the fluorescence polarization value (Figure 1). The amount of bound tracer is inversely proportional to the amount of free analyte in the sample, resulting in the polarization value inversely proportional to the concentration of the analyte [154].

Some immunoassay methods like ELISA require steps like washing multiple times or the separation of free from antibody-bound analyte. In FP technique, the time-consuming pre-analytical steps are not necessary [155]. FP immunoassay has been applied in determinations of various mycotoxins in food commodities, such as ZEA in maize [156], DON in wheat-based products [157], AFB_1_ in maize [158], and OTA in rice [155]. However, FP method has limited sensitivity and accuracy compared to HPLC. This is likely due to the cross-reactivity of antibodies towards other fungal metabolites and food matrix component [34].

#### 3.3.3. The Aggregation-Induced Emission

**Figure 2:**
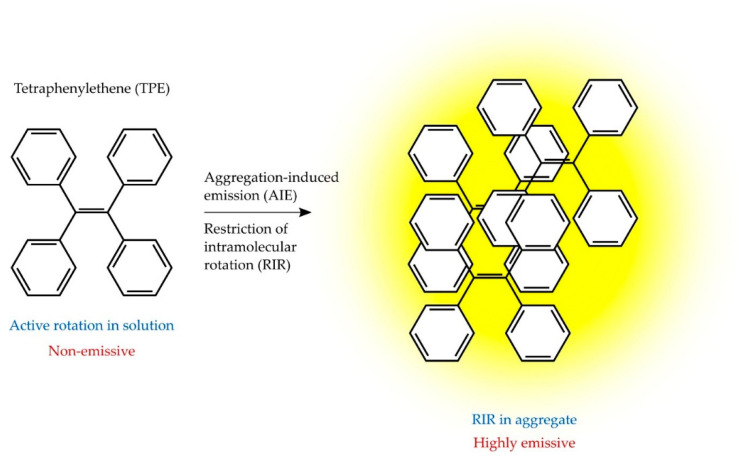
Scheme of the principle of aggregation-induced emission.

The aggregation-induced emission (AIE) is a photophysical phenomenon, in which a group of fluorescent dyes glows faintly in the dilute solution state, while their fluorescence is notably enhanced in the aggregation state (Figure 2) [159]. Intense dyes’ fluorescence may be the result of restricted intramolecular rotations in the aggregate state [160].

AIE dyes, which show high fluorescence emission in the aggregate states, are 9,10-distyrylanthracene (DSA), silacyclopentadiene (silole), tetraphenylethene (TPE), and their derivatives [161]. AIE dye-based aptasensor has been successfully developed for OTA detection in wine and coffee [159] and AFB_1_ in peanut oil and broad bean sauce [162].

#### 3.3.4. Molecularly Imprinted Polymers

**Figure 3:**
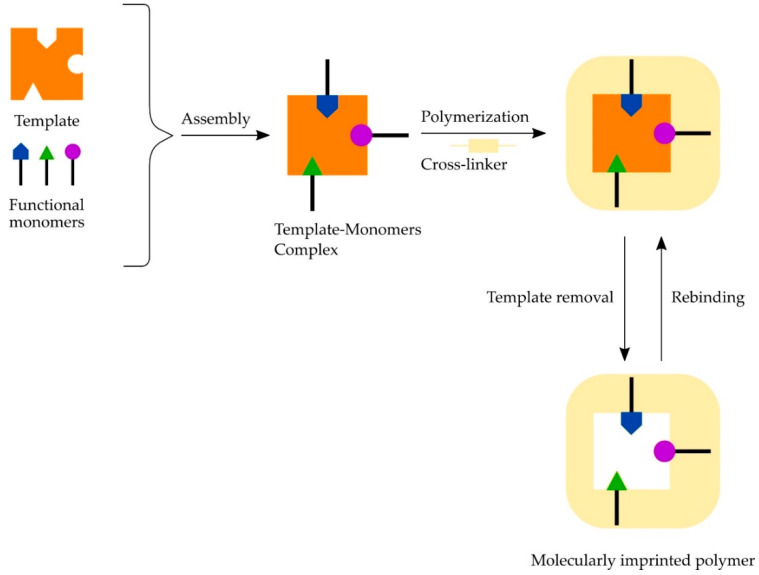
Scheme of molecularly imprinted polymer preparation.

Molecularly imprinted polymers (MIPs) is a synthetic method, which is designed to mimic natural recognition entities like antibodies and biological receptors with specificities similar to antibody-antigen interactions (Figure 3) [163]. During molecular imprinting, cross-linked polymers are formed by free-radical co-polymerization of functional monomers and a cross-linker in the presence of an analyte (like mycotoxins) serving as template [164].

The advantages of MIP are primarily their high selectivity and affinity for the target molecule used in the imprinting procedure, their resistance, raised temperature and pressure, inertness to bases, acids, metal ions, and organic solvents. Moreover, their synthesis costs are low, the storage time can be very long, and the MIPs keep their recognition capacity for several years at room temperature [163,165]. MIPs have been developed for the analysis of AFB_1_ in wheat [166], OTA in beer and wine [167], ZEA in cereals [168,169], and offers a great potential for further development in mycotoxins’ detection [34].

## 4. Conclusions

Contamination of agricultural products by mycotoxins resulted in establishing their acceptable limits in food and in the development of sensitive and effective detection methods. A significant step in mycotoxin analysis is sample preparation and different extraction followed by purification protocols. The best extraction techniques should use a small amount of chemical solvent, good extraction efficiency, and be relatively fast. Although many analytical methods are continuously optimized and validated and many novel methods are still being developed, chromatographic techniques, especially the LC/MS-MS technique are an essential tool for the detection of numerous mycotoxins. Chromatographic techniques ensure high sensitivity and reliability, as well as enable the simultaneous detection of different mycotoxins, regardless of their chemical structure and biological activity. However, if mobility is needed and rapid on-site analysis is required, for example at a food production site, the use of immunoassay-based methods such as LFIA is a good option. In contrast to chromatographic techniques, no qualified personnel are needed, the tests are simple to use, and the costs are low. Recent advances in detection and analysis technology and the development of novel techniques such as electronic nose, aggregation-induced emission, fluorescent polarization, or molecularly imprinted polymers reveal new possibilities in mycotoxin determination and may, in the future, constitute additional or independent detection and analysis techniques.
